# Molecular characterization and phylogenetic studies of *Echinococcus granulosus* and *Taenia multiceps* coenurus cysts in slaughtered sheep in Saudi Arabia

**DOI:** 10.1515/biol-2021-0131

**Published:** 2021-11-27

**Authors:** Jamila S. Al Malki, Nahed Ahmed Hussien

**Affiliations:** Department of Biology, College of Science, Taif University, P.O. Box 11099, Taif 21944, Saudi Arabia

**Keywords:** *Echinococcus granulosus*, *Taenia multiceps*, coenurus, *COX*1, *NAD*1, Taif, Saudi Arabia

## Abstract

Taeniids, consisting of two genera *Echinococcus* and *Taenia*, are obligatory tapeworms of mammals, and their pathogenicity was due to infection with larval stages. Hydatid (the larval stage of *Echinococcus granulosus*) and coenurus (the larval stage of *Taenia multiceps*) cysts are prevalent in domestic, wild ruminants, livestock, swine, and dogs, and accidentally they could also be found in humans. They lead to different clinical manifestations that cause economic loss in livestock and human morbidity. In Saudi Arabia, few studies were performed on hydatid and coenurus cyst genetic variations. The main goal of the present study was to identify *E. granulosus* and *T. multiceps* cyst isolates collected from slaughtered Harri sheep in Saudi Arabia by partial sequencing with PCR amplification of the cytochrome C oxidase 1 (*COX*1) gene. Molecular and phylogenetic evaluation based on *COX*1 sequences indicated that cyst isolates belong to *E. granulosus* and *T. multiceps*, respectively, successfully submitted in NCBI Genbank. Molecular characterization showed a low nucleotide diversity with two submitted isolates of coenurus with related isolates of Genbank. Conversely, *E. granulosus* isolates showed higher nucleotide diversity. The reported data could serve as a foundation for future molecular epidemiological and biological studies.

## 1 Introduction

Taeniid tapeworms belong to subclass Eucestoda, order Cyclophyllidea, and family Taeniidae, and they represent zoonotic parasites of mammals and livestock. Family Taeniidae consists of two genera, *Echinococcus* and *Taenia*, and they are mainly focused on for their socioeconomic impact by causing human morbidity and loss of livestock [1,2].

*Echinococcus granulosus* causes hydatidosis disease due to its larval stage infection. Hydatidosis (cystic echinococcosis) is widely distributed, and its prevalence varies according to climate and their contact with livestock [3]. Hydatid cysts have been reported in different livestock, including sheep, camels, cattle, and goats, with various incidence rates, resulting in significant economic losses [4,5,6]. Recently, it was recorded that the monetary burden of surgically treated human cystic echinococcosis increased that differs from one country to another [7]. In addition, it was reported that about 2.5% of humans infected with hydatidosis died after resorting to surgery [8]. There are three possibilities for cystic echinococcosis management: medical treatment with albendazole, percutaneous procedures, and surgery that depends on cyst stage, state, and organ location [9].

Coenurus represents the infective larval stage of *Taenia multiceps*; they are also widely distributed mainly in the tropical countries in Africa, Asia, and the Middle East [10]. *T. multiceps* are found as an obligate intestinal tapeworm of dogs, also commonly found in sheep and goats, as an intermediate host for the parasite, but the infection was extended to cattle and humans [11]. Mainly, coenurus cysts present in the brain in either acute or chronic form also could be frequently found in intramuscular and subcutaneous tissues [12,13,14]. Coenurosis leads to different clinical manifestations according to the location and extent of the coenuri. Coenurus cysts in the brain cause fever, muscle tremors, hemorrhagic retinal lesions, paralysis, ataxia, blindness, nystagmus, dysmetria, and scoliosis [15,16]. However, coenuri cysts in the subcutaneous and intramuscular tissues damage the functioning of normal organs and lead to muscular pain [11].

Due to the veterinary and medical significance of both *E. granulosus* and *T. multiceps*, there has been an intensive focus on their ecological, epidemiological, and taxonomic studies. Molecular characterization among many species found within Taeniidae, especially for the genus *Echinococcus*, has been well documented, but this was scarce with coenurus. However, there are very few documented studies on genetic variability regarding Taeniids in Saudi Arabia.

The present study aimed to (i) genetically characterize hydatid (larval stage of *E. granulosus*) and coenurus (larval stage of *T. multiceps*) cysts isolated from slaughtered Harri sheep in Taif, Saudi Arabia, by using mitochondrial cytochrome C oxidase 1 (*COX*1) and NADH dehydrogenase subunit 1 (*NAD*1) genes. Also, the study aimed to (ii) report the genetic relationship between selected genes loci of Taif isolates and other isolates present in other countries in Genbank.

## 2 Material and methods

### 2.1 Sample collection

The present study was carried out on Harri sheep from an animal slaughterhouse in Taif governorate (21.2819°N, 40.3841°E), Mecca Province, Saudi Arabia. During postmortem veterinary examination, meat was systematically inspected for hydatid and coenurus cysts occurrence by applying procedures of the routine meat inspection (sampling period from January to October 2020). Hydatid cysts were found in muscles, liver, and viscera, but coenurus cysts were found only in muscles. Therefore, we have collected samples from 20 sheep (gender, female; age, 1–2 years), about 200–300 g of muscles from each animal, which contains either hydatid or coenurus cysts from slaughtered sheep under permission from the Ministry of Environment, Water and Agriculture (KSA). Each cyst from a different animal was labeled and handled as a different isolate before carrying out the examination.

### 2.2 Microscopic examination

Ten samples of hydatid cysts (of *E. granulosus*) and the other ten samples of coenurus cysts (of *T. multiceps*) were collected from animal meat and carefully opened with the help of a sterile scalpel. First, protoscolices of hydatid and coenurus cysts were scraped off the inner wall of their cysts. Then, protoscolices were loaded on glass slides, covered, and examined under a light microscope without staining.

### 2.3 DNA extraction of protoscolices

About 300 µL of lysis buffer TNES (10 mM Tris, 400 mM NaCl, 100 mM Na_2_EDTA, 0.6% SDS, pH 7.5) was added to hydatid protoscolices in a microtube; then samples were frozen (in liquid nitrogen) and thawed (5×). To facilitate sample digestion, samples were ground before proteinase K (8 µL, 20 mg/mL) and incubated overnight at 37°C. Next, phenol/chloroform (100 µL) was added to the sample and centrifuged (16,500 g) for 5 min, and then, the clear upper phase was transferred into a new 1.5 mL Eppendorf. Next, for DNA precipitation, the same volume of absolute ethyl alcohol and sodium acetate (1%, 3 mol) was added to the sample and stored overnight (−20°C). Finally, samples were centrifuged (16,500 g) for 5 min, the supernatant was discarded, the pellet containing DNA was left to dry, and the pellet was finally dissolved in 100 µL sterile deionized water [17]. DNA was extracted from coenurus protoscolices with the same previous procedure using TNES lysis buffer and phenol/chloroform, but there was no need for freezing, thawing, or grinding.

### 2.4 PCR amplification

We have targeted two mitochondrial *COX*1 and *NAD*1 regions of *E. granulosus* and *T. multiceps* cysts by using two different primers JB3: 5′-TTTTTTGGGCATCCTGAGGTTTAT-3′, JB4.5: 5′-TAAAGAAAGAACATAATGAAAATG-3′ and JB11 5′-AGATTCGTAAGGGGCCTAATA-3′, JB12: 5′-ACCACTAACTAATTCACTTTC-3′, respectively [17]. The PCR reaction was set up with initial denaturation at 95°C (5 min), 40 cycles of denaturation at 94°C (45 s), primer annealing at 51°C (*COX*1) or 55°C (*NAD*1) (35 s), and then primer extension at 72°C (45 s). Final extension at 72°C (10 min) was necessary for complete amplification (Programmable Thermal Cycler, PTC-100TM thermal cycler, Model 96; MJ Research, Inc., Watertown, MA). PCR products were separated on 1% tris-borate/EDTA agarose gels and ethidium bromide staining and then visualized under gel documentation (Bio-Rad, USA) [18].

### 2.5 Sequencing and phylogenetic evaluation

Different PCR products of *COX*1 and *NAD*1 of *E. granulosus* and *T. multiceps* cysts were randomly selected and subjected to sequencing using an ABI Prism 3730 Genetic Analyzer automated sequencer. Four sequences of *COX*1 regions (two of each hydatid and coenurus cysts) were directly submitted in NCBI Genbank to have accession numbers. Sequences of *COX*1 and *NAD*1 were aligned with different sequences in Genbank using online NCBI BLAST, phylogenetically estimated, and finally viewed as rectangular cladogram in the phylogenetic Tree View [19].

## 3 Results

### 3.1 Morphological and microscopic examination of cysts

**Figure 1:**
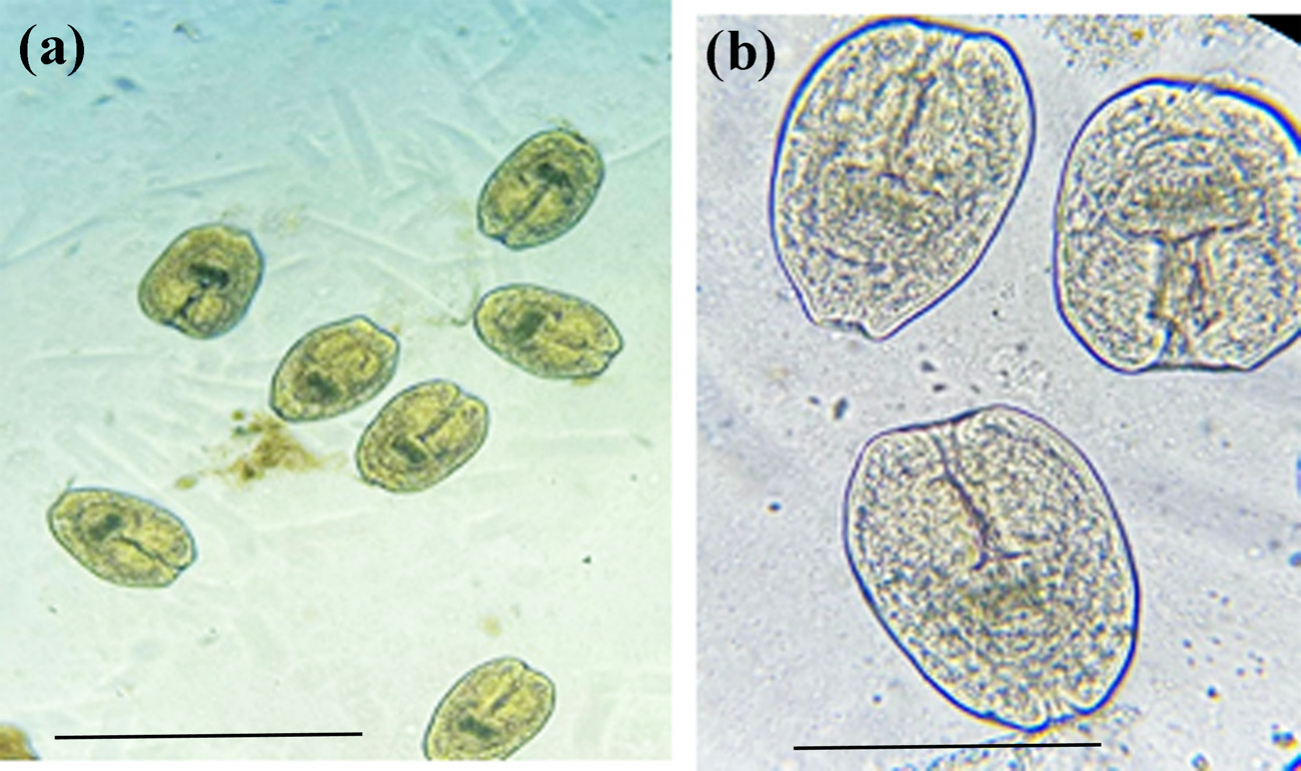
*Echinococcus granulosus* protoscolices at 10× (a), and 40× (b) magnifications. Scale-bars: 100 µm.

**Figure 2:**
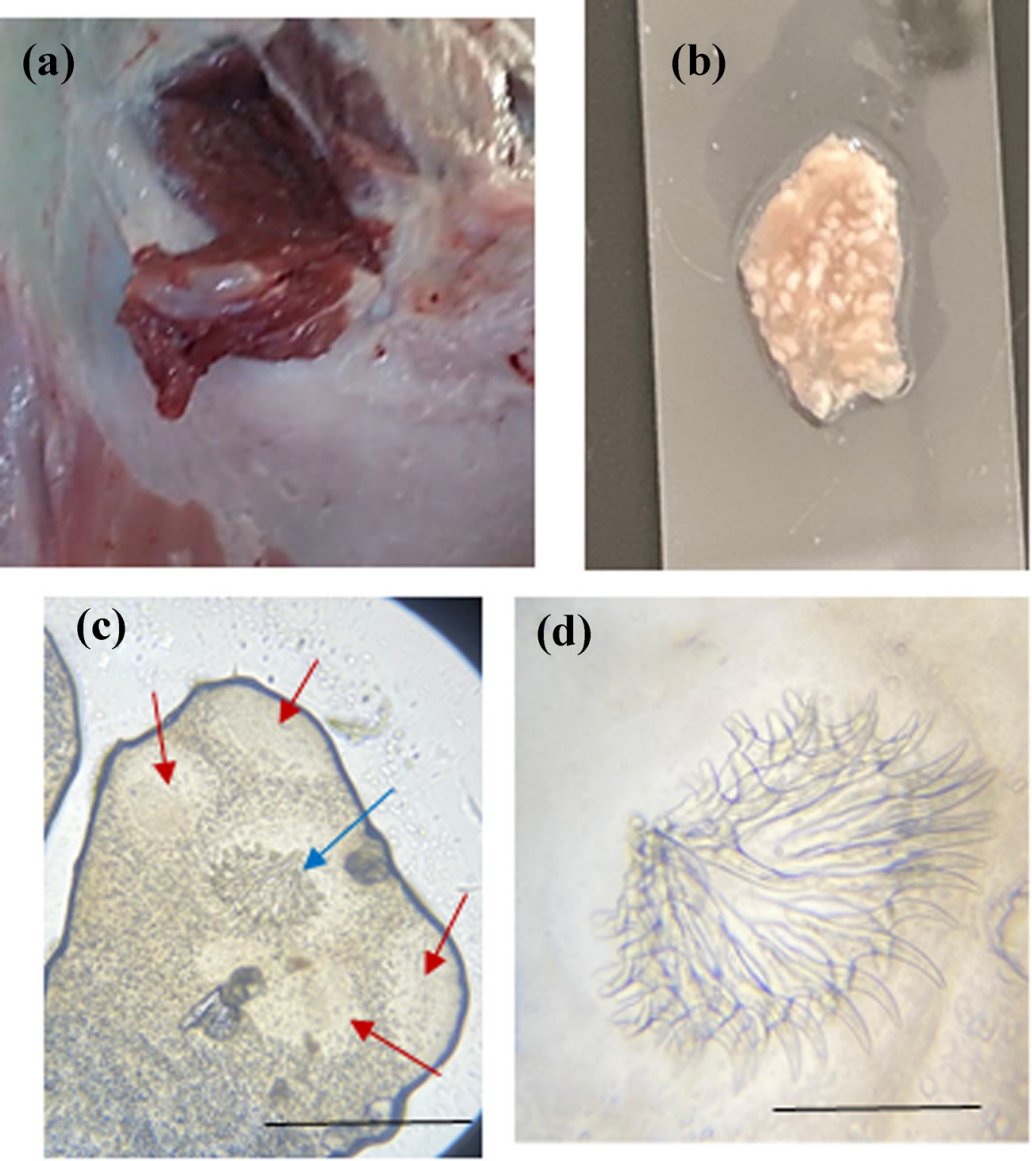
(a) Coenurus cyst attached to a muscle. (b) Multiple scolices appeared after cyst opening. (c) Isolated protoscolex large and small rostellar hooks: blue arrow and 4 suckers: red arrows. (d) Isolated rostellar hooks. Scale bars: 100 µm.

Hydatid and coenurus cysts appeared as fluid-filled round/oval sacs with different sizes embedded in the muscles of sheep. Protoscolices of both cysts were carefully scraped off the inner wall of their cyst rather than collecting them from cysts’ inner fluid. *E. granulosus* protoscolices appeared as an oval body with an invaginated scolex provided with several hooks (Figure 1). Visual examination showed numerous scolices in the inner wall of coenurus cysts. Microscopically, the isolated protoscolex showed both large and small rostellar taeniid hooks and four scup-shaped suckers (Figure 2).

### 3.2 PCR, sequencing, and phylogenetic analysis

**Figure 3:**
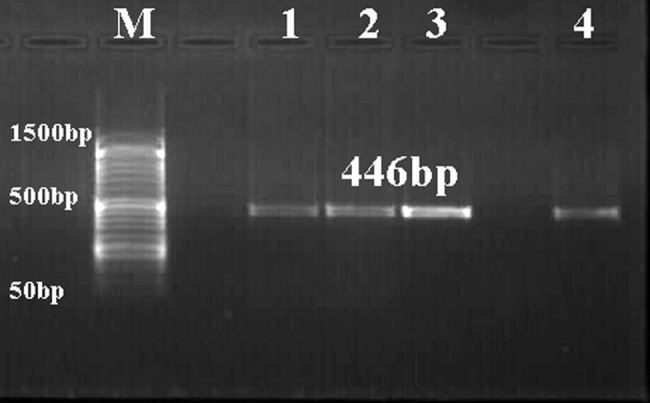
Representative agarose gel stained with ethidium bromide (1.5%) showing PCR product (446 bp) of COX1 of *E. granulosus* (lanes 1 and 2) and *T. multiceps* (lanes 3 and 4) cysts. Lane M: low-molecular-weight marker (50–1,500 bp).

**Figure 4:**
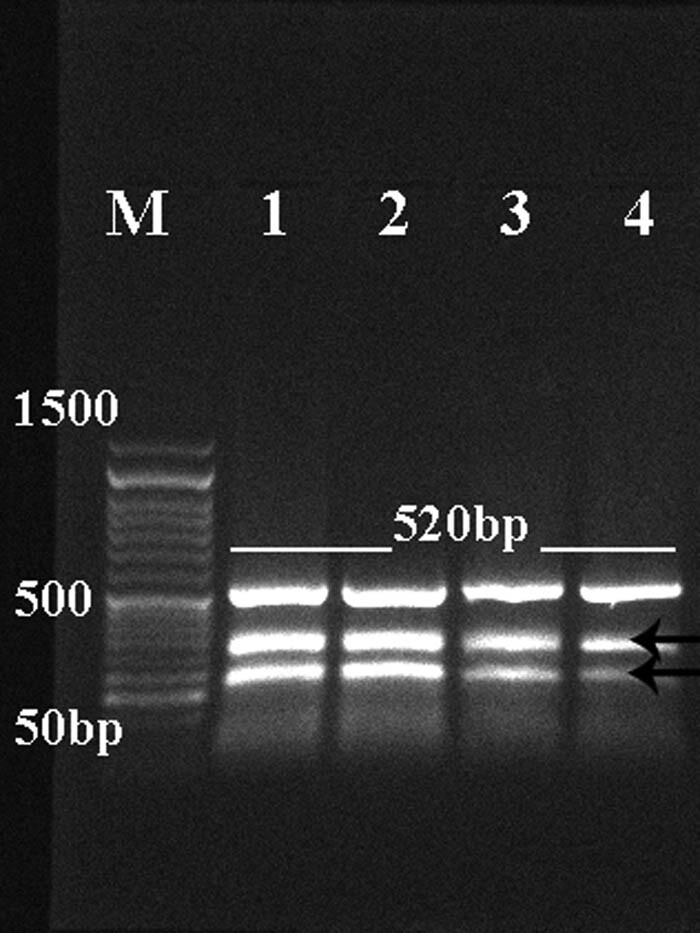
Representative 1.5% agarose gel showing PCR product (main amplicon size 520 bp) of NAD1 of *E. granulosus* (lanes 1 and 2) and *T. multiceps* (lanes 3 and 4) cysts. Low bands are nonspecific bands. Lane M: low-molecular-weight marker (50–1,500 bp).

Mitochondrial DNA was used to amplify two separate gene loci, *COX1* and *NAD1*, for both *E. granulosus* and *T. multiceps* cysts to yield amplicons of 446 bp and 520 bp, respectively. The present results report the success of PCR amplification for both selected portions (Figures 3 and 4). However, *NAD*1 amplification shows other lower nonspecific bands.

**Figure 5:**
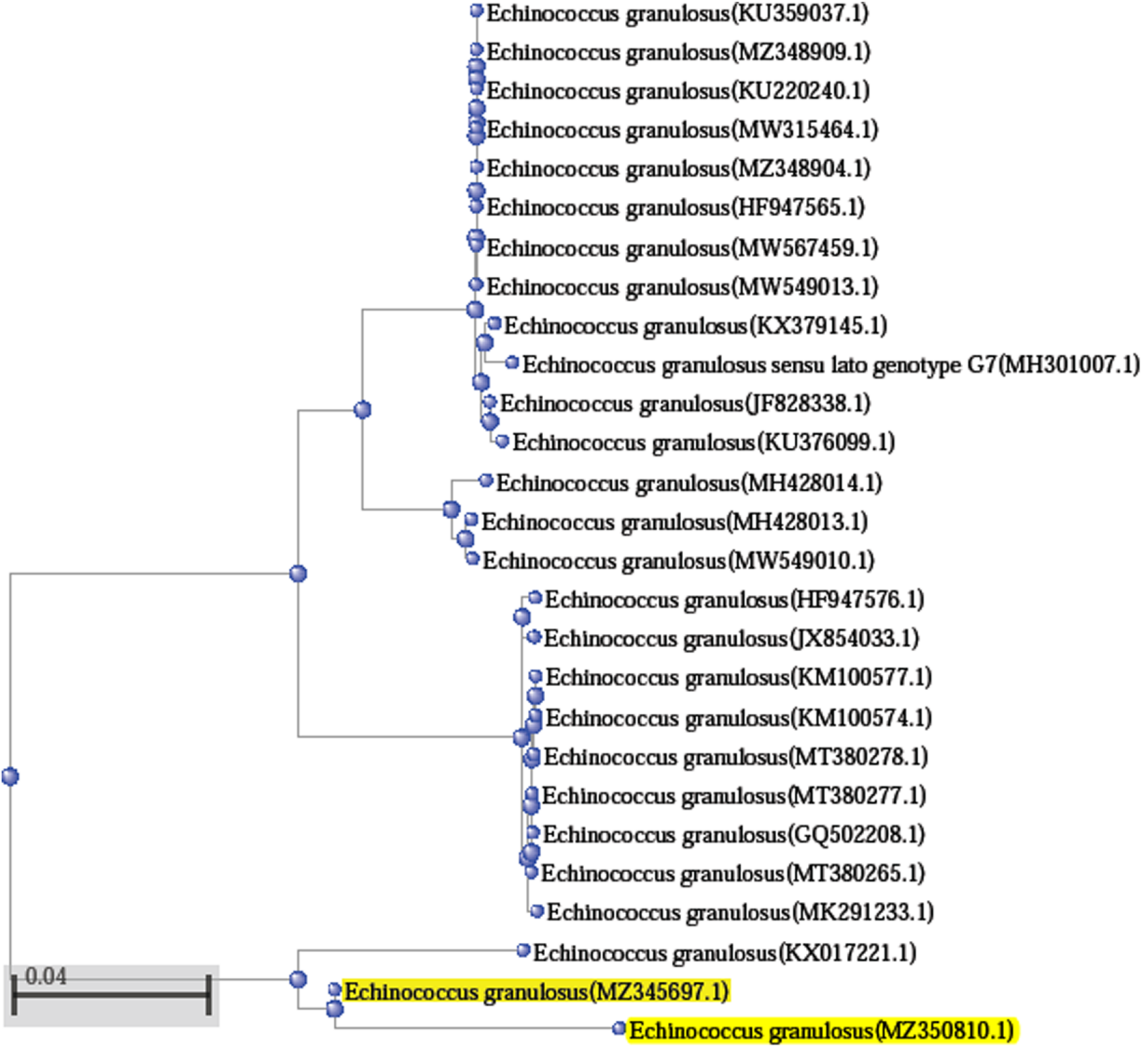
Phylogenetic relationships between Taif_Sheep *E. granulosus* (*COX1*) MZ345697.1 and MZ350810.1 (yellow highlights) with other reference sequences of *E. granulosus* from NCBI GenBank. GenBank sequences were shown by their name and accession numbers. Bar scale represents 0.05 nucleotide substitution per site.

**Figure 6:**
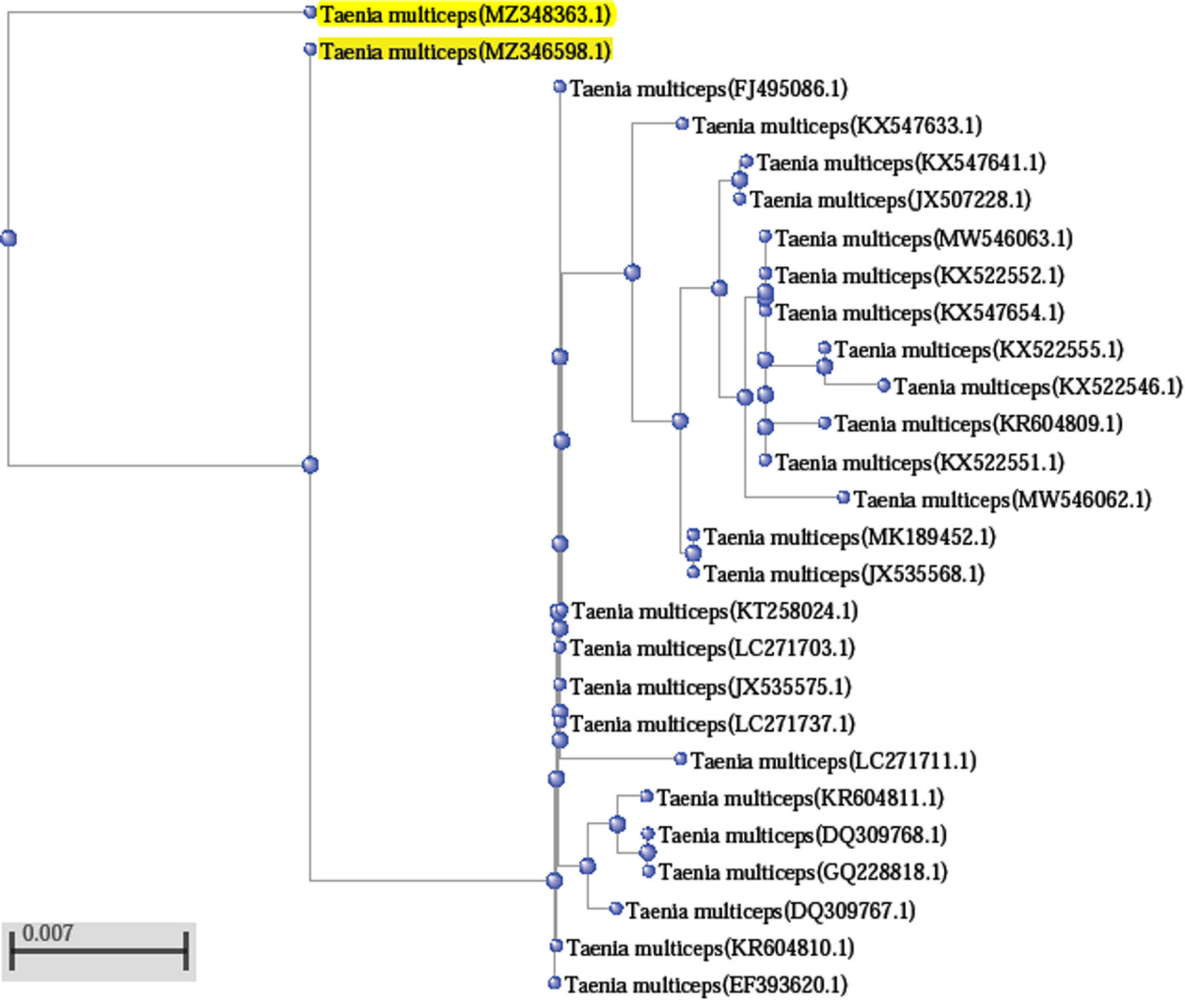
Phylogenetic relationships between Taif_Sheep coenurus cyst of *T. multiceps* (COX1) MZ346598.1 and MZ348363.1 (yellow highlights) with other reference sequences of *T. multiceps* from NCBI GenBank. GenBank sequences were shown by their name and accession numbers. Bar scale represents 0.007 nucleotide substitution per site.

Randomly selected PCR products from the two loci were sequenced by using their forward/reverse primers. Sequences of *COX1* were deposited in Genbank and have been assigned different accession numbers: *E. granulosus* (*COX*1) MZ345697.1 and MZ350810.1; and *T. multiceps* coenurus cyst (*COX*1) MZ346598.1 and MZ348363.1, respectively. MZ345697.1, MZ350810.1, MZ346598.1, and MZ348363.1 were blasted with other related sequences in different countries. Phylogenetic trees were constructed from those sequences according to higher percentage identity and query coverage range, as shown in Figures 5 and 6. Sequences of *NAD1* could not be blasted with other sequences nor submitted in Genbank due to the presence of nonspecific bands in PCR products that lead to the high noisy background in FASTA sequence (data not shown).

For *E. granulosus* (*COX*1): The BLAST analysis showed that Taif sheep isolate of *COX*1 sequence exhibited percentage identity 84–95.67% with Query coverage ranging from 94 to 100% of *E. granulosus* in GenBank isolates that were collected from other different countries. Alignment of MZ345697.1 with the other isolate MZ350810.1 showed identity with 94.95%. MZ345697.1 showed a higher alignment identity (95.67%) with *E. granulosus* KX017221.1 from dog stool in Palestine. MZ345697.1 showed 85% homology with Iran isolates KU359037.1, MW549010.1, MW567459.1, MW315464.1, MW549013.1, KU220240.1, and KU376099.1 from different hosts such as camel, sheep, and goat. MZ345697.1 showed 85% identity with *E. granulosus* isolates from Egypt MZ348909.1 and MZ348904.1 in camel and *Homo sapiens*, respectively. In addition, the *COX*1 isolate of Taif sheep showed the same identity percentage with other isolates from buffalos (MH428014.1 and MH428013.1) in India, cattle (HF947565.1) in Portugal, goat (KX379145.1) in Italy, pig (JF828338.1) in Brazil, and sheep (GQ502208.1) in Chile (Figure 5). MZ345697.1 showed lower homology (84%) with isolates from cattle in Turkey (KM100577.1, KM100574.1, MT380278.1, MT380277.1, and MT380265.1), Portugal (HF947576.1), *Homo sapiens* in India (JX854033.1), Iran (MK291233.1), and *Homo sapiens* in Poland (MH301007.1).

For larval stage (coenurus) of *T. multiceps* (*COX*1): The BLAST analysis showed that *COX*1 sequence (Taif-sheep isolate) exhibited a higher percentage identity 99–98% (Query coverage 100–97%) with that of *T. multiceps COX*1 in GenBank isolates that were collected from other different countries. Taif isolate 1, MZ346598.1, showed 97% homology with our second isolate, MZ348363.1, in the present study. MZ346598.1 showed a higher alignment identity (99%) with coenurus cyst isolates found in brains of sheep in Egypt (LC271737.1 and LC271703.1), isolates from sheep and humans in China (JX535575.1, FJ495086.1, and KT258024.1), Greece (KR604810.1), and Turkey (EF393620.1). In addition, MZ346598.1 showed 98% identity with coenurus (larval stage of *T. multiceps*) isolates from different hosts of China (KX547633.1, KX547641.1, MW546063.1, KX522551.1, KX547654.1, KX522552.1, JX535568.1, GQ228818.1, and JX507228.1), from goat brain, dog intestine, goat muscle, and brain of domestic yak. Moreover, MZ346598.1 showed 98% homology with coenurus *COX*1 isolates from Italy (DQ309767.1 and DQ309768.1), Greece (KR604811.1), and Egypt (LC271711.1). Lower identity percent was found in MZ346598.1 alignment (97%) with isolates from China (KX522555.1, MW546062.1, and KX522546.1) and Greece (KR604809.1).

## 4 Discussion

Cystic echinococcosis and coenurosis are zoonotic diseases that affect humans and livestock due to infection with the larval stages of *Echinococcus granulosus* and of *Taenia multiceps* cysts, leading to economic loss that increases annually [4,9,11]. Dogs, especially stray unvaccinated dogs, are definitive hosts for different cestodes of Taeniidae family, including *E. granulosus* and *T. multiceps*. Dogs were commonly associated with extensive livestock herding to protect domestic animals against predators that could lead to their infection [20,21]. For the first time, the present study reports the genetic characterization of two different parasite cysts, hydatid (the larval stage of *E. granulosus*) and coenurus (the larval stage of *T. multiceps*), in Taif governorate, Makkah Province, Saudi Arabia.

Sheep, goats, cattle, and camels represent the primary livestock species producing red meat in Saudi Arabia, with an estimated total population of 13,444,435 heads [22]. Sheep represent the significant population (72%) of the livestock, and they import large numbers of sheep to satisfy the needs of Saudi citizens. Awassi, Harri, and Najdi are the most available sheep species in Saudi Arabia [23]. Any endemic infection for livestock, such as hydatidosis and coenurosis, could lead to economic setbacks; therefore, there is a need to highlight their study, especially in KSA.

Hydatidosis and coenurosis due to *E. granulosus* and *T. multiceps* larval infection, respectively, cause significant damage in livestock that leads to production losses, especially in endemic areas [2,24]. Moreover, these parasites were distributed worldwide in different intermediate hosts, especially in sheep and goats, accidentally humans, reflecting their medical and veterinary importance [25].

The present study aims to identify two Taeniids parasites found in slaughtered Harri sheep meat based on *COX*1 and *NAD*1 partial sequencing and phylogenetic relationship. It was reported that the development of molecular techniques rather than traditional morphological criteria has provided improved tools for taeniid species identification and investigating relationships among them. Sequencing of mitochondrial DNA, especially *COX*1 and *NAD*1 loci, has been successfully used for molecular characterization and identification of taeniids tapeworm [26,27].

Our choice to use mitochondrial DNA (mtDNA) sequence, especially *COX*1 and *NAD*1, for molecular characterization of *E. granulosus* and *T. multiceps* larva was based on the previous assessment. mtDNA is widely used in molecular characterization and phylogenetic evaluation studies. *COX*1 gene represents the most common gene of mtDNA for phylogenetic analysis and evolutionary biology of helminth parasites to determine interspecific and/or intraspecific variation [8,28,29,30]. The mitochondrial *COX*1 gene is very suitable for genetic diversity detection and haplotype analysis. Its evolutionary change rate is slow enough for the same species but fast enough for differentiation between different species. Therefore, the mitochondrial *COX*1 gene has been selected for use in the creation of DNA barcodes and species differentiation of different helminths as our present study [31,32].

Worldwide, there have been well-documented research studies about the genetic variability among many species found within the Taeniidae, especially for the genus *Echinococcus*. Still, there are a few related species of *Taenia*, especially for *T. multiceps* [33]. However, there is a scarcity of studies related to the genetic characterization of *E. granulosus* and *T. multiceps* larval stages in KSA and their phylogenetic relationship.

In the current study, a genetically characterized sample based on *COX*1 isolated from a hydatid cyst of MZ345697.1 showed identity about 94.95% with the other isolate MZ350810.1. For coenurus cyst, isolate1 MZ346598.1 showed 97% homology with the second isolate MZ348363.1. In addition, comparing partial sequences of Taif *COX*1 isolates from both cysts showed different identities with other isolates from organs present in various hosts found in other countries. The present results are inconsistent with previous studies, in which Al-Hizab et al. [34] and AL-Mutairi et al. [35] have reported *E. granulosus* species variation based on molecular characterization in infected sheep and camels in the Arabian Peninsula and KSA. AL-Mutairi et al. [35] have reported the phenotypic and genetic characterization of hydatid cysts isolated from sheep and camel meat in Al-Madinah, KSA. Genetic characterization of *E. granulosus* was based on random amplified polymorphic DNA polymerase chain reaction (RAPD-PCR) for the whole genome, PCR amplification of *COX*1 and 12S rRNA genes followed by single-stranded conformation polymorphism (SSCP), and then sequencing. They have investigated that cyst isolates slightly vary from each other and other isolates found in Genbank. They have concluded that there is an intraspecific variation in *E. granulosus* found in camels and local sheep.

In addition, Christodoulopoulos et al. [36] have investigated *COX*1 nucleotide diversity of coenurus cyst isolates from sheep and goats in Pakistan, and their phylogenetic analysis shows high related homology with China isolates but highly different from other isolates from different countries. They have reported that coenurus cerebralis commonly found in the brain (cerebral form) could also be found in other non-cerebral tissue such as intramuscular and subcutaneous tissues.

Diversity in the nucleotide sequences of the presently studied isolates was recorded before within the same region, such as in Iran [5,13], China [26], and Italy [37,38]. Most of their phylogenetic analysis depends on one or more mtDNA sequences such as nad1, cox1, and 12S rRNA. They have concluded that there are no monophyletic groups based on the intermediate host species, the organ from which the parasite was isolated, and geographical origin [33,36]. However, reporting parasite characterization and genetic identification will be crucial for controlling and preventing parasitic infections [8].

The limitation of this study is the use of the limited sample size, and only partial sequencing was done for the *COX*1 gene that could have implications on data interpretation. Thus, we recommend future studies considering a larger sample size, complete sequencing *COX*1, and other characterization-related genes in a massive epidemiological survey for further evaluation.

## 5 Conclusion

The present study reports the molecular characterization of hydatid (the larval stage of *E. granulosus*) and coenurus (the larval stage of *T. multiceps*) cysts present in slaughtered sheep meat in Taif, KSA. We have determined sequences differences within isolates of the same species that we submitted in GenBank and other isolates found in Genbank from different host organs in different countries. For a more precise identification and characterization of Taeniids in KSA, additional isolates from other hosts, other geographic areas, different molecular protocols, long-sequenced genes in the mitochondrial and nuclear genome, and further information concerning biological characteristics may be necessary to increase our understanding of the epidemiological distribution in KSA.
